# Health states for schizophrenia and bipolar disorder within the Global Burden of Disease 2010 Study

**DOI:** 10.1186/1478-7954-10-16

**Published:** 2012-08-22

**Authors:** Alize J Ferrari, Sukanta Saha, John J McGrath, Rosana Norman, Amanda J Baxter, Theo Vos, Harvey A Whiteford

**Affiliations:** 1 The University of Queensland, School of Population Health, Herston, Queensland, Australia; 2 Queensland Centre for Mental Health Research, The Park Centre for Mental Health, Wacol, QLD, Australia; 3 The University of Queensland, Queensland Brain Institute, St Lucia, QLD, Australia; 4 The University of Queensland, Queensland Children’s Medical Research Institute, Herston, QLD, Australia

**Keywords:** Global Burden of Disease, Health States, Schizophrenia, Bipolar Disorder

## Abstract

A comprehensive revision of the Global Burden of Disease (GBD) study is expected to be completed in 2012. This study utilizes a broad range of improved methods for assessing burden, including closer attention to empirically derived estimates of disability. The aim of this paper is to describe how GBD health states were derived for schizophrenia and bipolar disorder. These will be used in deriving health state-specific disability estimates. A literature review was first conducted to settle on a parsimonious set of health states for schizophrenia and bipolar disorder. A second review was conducted to investigate the proportion of schizophrenia and bipolar disorder cases experiencing these health states. These were pooled using a quality-effects model to estimate the overall proportion of cases in each state. The two schizophrenia health states were acute (predominantly positive symptoms) and residual (predominantly negative symptoms). The three bipolar disorder health states were depressive, manic, and residual. Based on estimates from six studies, 63% (38%-82%) of schizophrenia cases were in an acute state and 37% (18%-62%) were in a residual state. Another six studies were identified from which 23% (10%-39%) of bipolar disorder cases were in a manic state, 27% (11%-47%) were in a depressive state, and 50% (30%-70%) were in a residual state. This literature review revealed salient gaps in the literature that need to be addressed in future research. The pooled estimates are indicative only and more data are required to generate more definitive estimates. That said, rather than deriving burden estimates that fail to capture the changes in disability within schizophrenia and bipolar disorder, the derived proportions and their wide uncertainty intervals will be used in deriving disability estimates.

## Background

Considerable progress has been made over the last 15 years in establishing the extent to which mental disorders as a group and individual disorders contribute to disease burden. The Global Burden of Disease (GBD) study has been influential in quantifying these contributions [1]. The first GBD study used disability-adjusted life years (DALYs) to provide gender- and age-specific burden estimates for over 100 diseases, injuries, and risk factors in eight regions of the world in the year 1990 [2]. Such estimates of burden have been used in a range of public health contexts such as the prioritization of governmental funding in health service delivery and research [3-6]. Refinement of the DALY methodology, for example the derivation of social preferences such as the disability weights, as well as the epidemiological data used in DALY calculations, is necessary to ensure that burden estimates are as accurate as possible [7].

While the World Health Organization (WHO) has provided interim updates of GBD estimates since 2000 for the world and 14 regions, a new GBD study (GBD 2010 Study) is expected to be completed by 2012 [8]. The GBD 2010 Study utilizes improved methods for assessing burden [3] and will provide estimates of burden for 1990, 2005, and 2010. One area of refinement concerns the nonfatal component of DALYs, i.e., the years of life lived with a disability (YLD). There has been much debate surrounding the manner in which disability is operationalized [9]. The literature on summary measures of population health uses the concept of “health states” in their definition of disability. Disability is understood in terms of individuals with a disorder spending time in one or more health states associated with that disorder [9].

What constitutes a health state and how it should be measured will depend on the summary measure being used and its function [9]. For GBD purposes, a principle of “treating like health outcomes as like” is assumed when defining health states. This contends that a particular disease will result in health states with the same level of disability across multiple settings [10]. Health states in the DALY methodology are restricted to “within the skin” elements of functioning such as body functions, senses, cognition, and ambulation. This restriction is chosen in order to compare the relative magnitude of decrements in health across diseases, countries, and time [1,9,10]. “Out of skin” elements of functioning such as participation restrictions, while important, are beyond the scope of what the DALY aims to capture [10,11].

Within GBD methodology, the severity of each health state is quantified using a disability weight. This allows estimates of disease burden to capture differences in disability both between and within disorders [11,12]. In the GBD 2010 Study, disability weights are being estimated for around 230 health states [1,13] using two different sources of data. A population-based survey has been conducted in Bangladesh, Indonesia, Peru, Tanzania, and the United States of America supplemented by an Internet survey (gbdsurvey.org) to access as wide a range of views as possible [12,13].

The purpose of this paper is to describe GBD health states for schizophrenia and bipolar disorder. This involves (1) presenting clinical definitions for each health state and (2) using these definitions to quantify the overall distribution of cases in each health state. Although related, this is a separate exercise to the formulation of lay descriptions of each health state included in the population- and Internet-based disability weight surveys mentioned earlier. Rather, the deliverables of this paper will inform the differences within the course of the disorder for which health state-specific disability weights are being developed. The distribution of schizophrenia and bipolar disorder cases across multiple health states can be summarized differently depending on the approach used. For instance, the number of cases within the population in each state or the mean or median time spent in each state may be reported. Since burden of disease estimates are cross-sectional measurements of health loss in a population, in a particular year, the former approach of measuring the number of cases in each health state fits best and will be used here.

## Methods

First a review of the literature was conducted to identify health states to describe schizophrenia and bipolar disorder. This was followed by a systematic review to identify epidemiological data on the severity distribution across health states. Finally, a meta-analysis was carried out to pool the proportion of cases of schizophrenia and bipolar disorder in each health state, across studies, for use in burden estimation in the GBD 2010 Study.

### Defining health states

#### Schizophrenia

There are a number of descriptions of schizophrenia in the literature that could be used to describe the health states found in this disorder. For GBD purposes health state definitions were based on a diagnostic system and general clinical experience, which converge on a small set of descriptors. The two schizophrenia health states for GBD are reminiscent of Crow’s [14,15] type I and type II schizophrenia [16-18], which has influenced the Diagnostic and Statistical Manual of Mental Disorders (DSM-IV-TR) [19].

The first health state, referred to as “acute”, was ascribed to schizophrenia with predominantly positive symptoms (e.g., delusions, hallucinations, and thought disorder). The second health state, referred to as “residual”, was ascribed to schizophrenia with predominantly negative symptoms (e.g., flat affect, loss of interest, and emotional withdrawal). These health states are not mutually exclusive. Positive symptoms can be followed by the development of negative symptoms or they can both occur simultaneously with one set of symptoms being more noticeable than the other [14,19], and this can be affected by treatment. Both positive and negative symptoms are associated with impaired functioning [20].

#### Bipolar disorder

Both the DSM-IV-TR and the International Classification of Diseases (ICD-10) describe bipolar disorder as the experience of one or more manic (or hypomanic) episodes and also one or more major depressive episodes [19,21]. The health state categories selected were based on this description. They were “manic”, “depressive”, and “residual”. A manic state involves elevated, expansive, or irritable mood. A depressive state involves depressed mood or loss of interest in daily activities. A residual state involves depressive or manic symptoms, which are below the threshold for a manic or depressive episode. A 20-year prospective study of bipolar I and II disorders showed that there were significant variations in levels of functioning depending on which health state the person was experiencing. Functioning was found to be equally poor during manic and depressive states except for a few instances when depression led to more disability. In comparison, functioning significantly improved when participants were less symptomatic (in a residual state), although functioning in this state was still significantly less than in controls [22].

### Epidemiological data on health states

Two separate systematic literature reviews were conducted to search for available epidemiological data on these health states for schizophrenia and bipolar disorder. For each review, the methodology used was in line with that proposed by the Meta-analysis Of Observational Studies in Epidemiology (MOOSE) Group [23].

#### Schizophrenia

For schizophrenia, relevant papers were accessed from an existing database of a systematic review of the prevalence of schizophrenia covering studies published between 1965 and 2002 [24]. Studies included in the analysis were those that reported the overall proportion of individuals with schizophrenia experiencing acute and residual states or provided sufficient data to calculate the necessary proportions.

#### Bipolar disorder

For bipolar disorder, a systematic literature search was conducted. A series of search strings were developed and used to search electronic databases such as Medline and PsycInfo. The literature search also involved reviewing reference lists and contacting experts in the field to obtain articles not identified through the database search. Studies included in the present analysis were those reporting the overall proportion of individuals with a bipolar disorder experiencing manic, depressive, and residual states or provided sufficient information to calculate the necessary proportions. See Additional file 1 for further information on the literature search and methodology.

Extracted data included information pertaining to the study methodology and sample (e.g., study design, sample ascertainment, location, age range) and the overall quality of the study (e.g., methodological quality, sample representativeness). Although current information (one month or less) from the time of survey was the gold standard, studies reporting longer ascertainment periods had to be included to maximize inclusion. A quality index was used to quantify the methodological quality of each study. Quality was rated based on variables describing key areas of the study methodology such as the ascertainment period, the representativeness of the sample, and the method of assessment. See Additional file 2 for more information on the quality index. The difference in proportions as a function of the economic status of the country from which the sample was drawn was also investigated. Estimates were categorized according to whether they were from developed, emerging, or developing economies, based on World Bank income categories [25,26].

### Analysis

MetaXL 1.0, a tool for meta-analysis in Microsoft Excel, was used to pool health state proportions from each study (see: http://www.epigear.com/). A “quality effects model” was chosen over the more conventional fixed or random effects models. This was to explicitly address heterogeneity in pooled proportions [27]. The standard in meta-analysis is to use the random effects model instead of a fixed effects model in the presence of significant heterogeneity. However, the random effects model assumes that the observed heterogeneity is driven by real differences in the distribution, when it is likely that it is also caused by differences in study quality. MetaXL implements a method to address study heterogeneity caused by differences in study quality. This quality effects model used quality scores from the systematic review to weigh studies not only according to sample size but also to study quality [28,29]. MetaXL ensures that the pooled proportions add up to one.

## Results

### Schizophrenia

Of the 188 studies included in the original systematic review for the prevalence of schizophrenia [24], only six studies met the inclusion criteria for the present review [30-35]. The three main reasons for exclusion were that health state definitions were not consistent with how they have been defined in this paper, that the data reported were not in the format required for GBD, and that only a subsample of the population was investigated (e.g., high risk groups). Key features of the six included studies are presented in Table 1.

**Table 1:** Summary of data from studies included for schizophrenia

**Reference**	**Country**	**Age range (years)**	**Number of cases**	**Quality Index Score (/1)**	**Percentage of acute cases**	**Percentage of residual cases**
Bondestam et al., 1990 [31]	Tanzania	15-99	10	0.7	60	40
Shen et al., 1981 [34]	China	15-99	341	0.5	51	49
Zharikov, 1986 [35]	Russia	N/S^a^	1429	0.3	50	50
Babigian, 1980 [30]	USA	0-99	3319	0.5	62	38
Fichter et al., 1996 [32]	Germany	18-99	7	0.8	75	25
Keith et al., 1991 [33]	USA	18-99	305	0.9	69	31
**Pooled health state proportions (%) (95% confidence intervals)**					**63%** (38%-82%)	**37%** (18%-62%)

Note. Values rounded up to nearest whole number; ^a^N/S: Not specified.

Studies were drawn from five different countries made up of three developing and emerging economies [31,34,35] and two developed economies [30,32,33]. Given the limited number of studies, the study from an emerging economy was combined with the two from developing economies. Based on the combined six studies, 63% (38%-82%) of schizophrenia cases were in an acute state and 37% (18%-62%) in a residual state (see Table 1). Although the pooled proportion of cases in an acute state was lower in the three developing/emerging countries compared with the developed countries, this effect was not statistically significant (54% (43%-65%) vs. 67% (46%-82%)). Since there were only three studies in each group, these estimates are indicative only, and more data would be required to make more definitive statements as to whether a difference exists.

### Bipolar disorder

Out of the 38 studies identified through the systematic literature review, six studies met criteria for inclusion [36-41]. The two main reasons for exclusion were that health state definitions were not consistent with how they have been defined in this paper and that the data reported were not in the format required for GBD. Key features of the six included studies are presented in Table 2.

**Table 2:** Summary of data from studies included for bipolar disorders

**Reference**	**Country**	**Age range (years)**	**Number of cases**	**Included diagnoses**^**a**^	**Quality Index Score(/1)**	**Percentage of manic cases**	**Percentage of depressive cases**	**Percentage of residual cases**
Morgan et al., 2005 [40]	Australia	18-64	112	BPI, BPII	1.0	19	20	62
Negash et al., 2005 [41]	Ethiopia	15-49	295	BPI	0.9	12	13	75
Cruz et al., 2008 [37]	European^b^	18-92	1563	BPI	0.9	11	11	79
Faravelli et al.,1990 [38]	Italy	15-99	6	BPI, BPII, BPNOS	0.8	33	50	17
Have et al., 2002 [39]	Netherlands	18-64	136	BPI, BPNOS	0.9	41	28	32
Blader et al., 2007 [36]	USA	5-64	3900	BPI, BPII, BPNOS	0.6	28	37	35
**Pooled health states proportions (%) (95% confidence intervals)**						**23%** (10%-39%)	**27%** (11%-47%)	**50%** (30%-70%)

Note. Values rounded up to nearest whole number; ^a^BP: Bipolar; NOS: Not otherwise specified; ^b^European: Belgium, Denmark, Finland, France, Germany, Greece, Ireland, Italy, Netherlands, Norway, Portugal, Spain, Switzerland, and the United Kingdom.

Five studies used samples from a different country each [36,38-41] and one study used a sample from multiple European countries (Belgium, Denmark, Finland, France, Germany, Greece, Ireland, Italy, Netherlands, Norway, Portugal, Spain, Switzerland, and the United Kingdom) [37]. Only one study provided estimates for a developing economy (Ethiopia) [41] and reported 12% of cases in a manic state and 13% in a depressive state. Based on all six studies, 23% (10%-39%) of bipolar cases were in a manic state, 27% (11%-47%) were in a depressive state, and 50% (30%-70%) were in a residual state (see Table 2).

## Discussion

The present review is a first attempt to systematically pool data on schizophrenia and bipolar health states for GBD. It revealed a lack of empirical data on the proportion of cases in the different health states. Since the field lacks readily operationalized criteria to describe schizophrenia and bipolar disorder health states, the choice of health states limited the data available. That said, definitions selected were made as representative as possible while remaining consistent to GBD methodology by using internationally accepted diagnostic systems of schizophrenia and bipolar disorder, which converge on a small set of descriptors.

Our definition of health states for both disorders conformed as much as possible to ‘within the skin’ elements of functioning required by GBD. However, the DSM diagnostic criteria and literature (particularly for schizophrenia) include both symptom severity and functional impairment, part of which are dependent on the environmental context. Consequently, trying to fit the multidimensional features of schizophrenia into two domains remains controversial. The fragility of the empirical data is a concern, particularly the lack of consensus on a health state definition and how the corresponding proportion of cases in each state is measured. For the purposes of this exercise, we have adopted a pragmatic approach in using the best available data to come up with a sensible set of health states (and health state proportions) that can be used in the GBD 2010 Study.

More data on health states were available for bipolar disorder than schizophrenia, which is likely to reflect more agreement in the literature on the health states that exist for bipolar disorder. Aside from estimating the number of cases in each state, which was the format used for GBD, some studies also estimated the time spent in each state. As discussed previously, data in this format were not included as they did not fit with GBD’s cross-sectional approach to quantifying burden.

In support of the present findings, Judd and collaborators’ 20-year prospective follow-up study investigating the weekly polarity of symptoms in patients with bipolar disorder showed that patients spent approximately three times more follow-up weeks in a residual state compared to a manic or depressive state [42]. This was also true in studies conducted by Joffe and collaborators [43], Kupka and collaborators [44], and Paykel and collaborators [45]. Our finding of almost equal numbers of cases in manic and depressive states, however, was not entirely consistent with literature based on expert opinion, which suggests that patients spend more time in a depressive state than a manic state. Further investigation into the alternative approaches to measuring the distribution of schizophrenia and bipolar disorder health states in the population and how these affect overall results (and ultimately burden estimates) is required for a better understanding of the above findings.

Due to paucity in the literature, studies using clinical samples were also included in this review. Although inpatient samples may not be representative of the general population, as most individuals will only be identified for inclusion once their illness is sufficiently severe to require hospital admission [46,47], clinical samples comprising both in- and outpatient samples are likely to be more representative where treatment rates are high. A mixture of inpatient and broader clinical samples were included in the present review. Studies included for bipolar disorder also differed in the subtype of bipolar disorder assessed. The study from Ethiopia [41] and the one conducted across multiple European countries [37] assessed bipolar I cases only, the latter of which also included cases of bipolar I disorder with rapid cycling. Although this was less representative of the spectrum of bipolar disorder, these studies were included to maximize the global distribution of the data.

There were insufficient data to consider the impact of treatment rates on the presentation of cases. It is expected that treatment alters the proportion of cases in each state. For instance, high treatment rates may lead to less disability associated with an illness with more cases in a residual state. Research in Western countries has found that the prognosis for psychotic disorders worsens with lack of treatment [48-50]. Evidence for this in non-Western countries is not as clear. There has been literature to suggest that patients with schizophrenia have better functioning in developing countries where treatment rates are low compared to in developed countries where it is high [51] and alternatively literature to suggest that individuals with schizophrenia in developing countries have significantly poorer prognosis than originally thought [52].

## Conclusions

Gaps in the literature meant that only a small selection of studies could be used to derive pooled proportions. Consequently, there may not have been enough data to yield a representative indication of proportions. Also, the data sources identified reported a range of methodological differences. This has led to considerable heterogeneity in the data. We expect that the inclusion criteria used and the quality effects model has controlled for some of this heterogeneity in the final pooled proportions, but the wide uncertainty intervals reflect the poor state of knowledge. Schizophrenia and bipolar disorder both have a chronic course, with fluctuations in symptomatology and disability [47,53,54]. Rather than deriving burden estimates that fail to capture the changes in disability within these disorders, we will use the data available to us to make more representative estimates of disease burden in the GBD 2010 Study.

## Competing interests

Core funding for the Queensland Centre of Mental Health Research is provided by the Queensland Department of Health. TV and RN have received funding from the Global Burden of Disease project.

## Authors’ contributions

AJF, SS, JJM, AJB, TV, and HAW contributed to the conceptualization of the paper. AJF and SS conducted the literature search. AJF wrote the first draft of the paper. RN provided statistical advice and contributed to the statistical design. AJF and RN completed the statistical analyses. All authors contributed to and have approved the final manuscript.

## Supplementary Material

Additional file 1 Literature search strategy for bipolar disorder health states. Click here for file

Additional file 2 Quality Index criteria. Click here for file
