# Supplementary figures and images for: Health states for schizophrenia and bipolar disorder within the Global Burden of Disease 2010 Study
Source: Popul Health Metr. 2012 Aug 22;10:16. doi: 10.1186/1478-7954-10-16 (PMC3490927; doi:10.1186/1478-7954-10-16)

## Additional file 1.

### Literature search strategy for bipolar disorder health states.

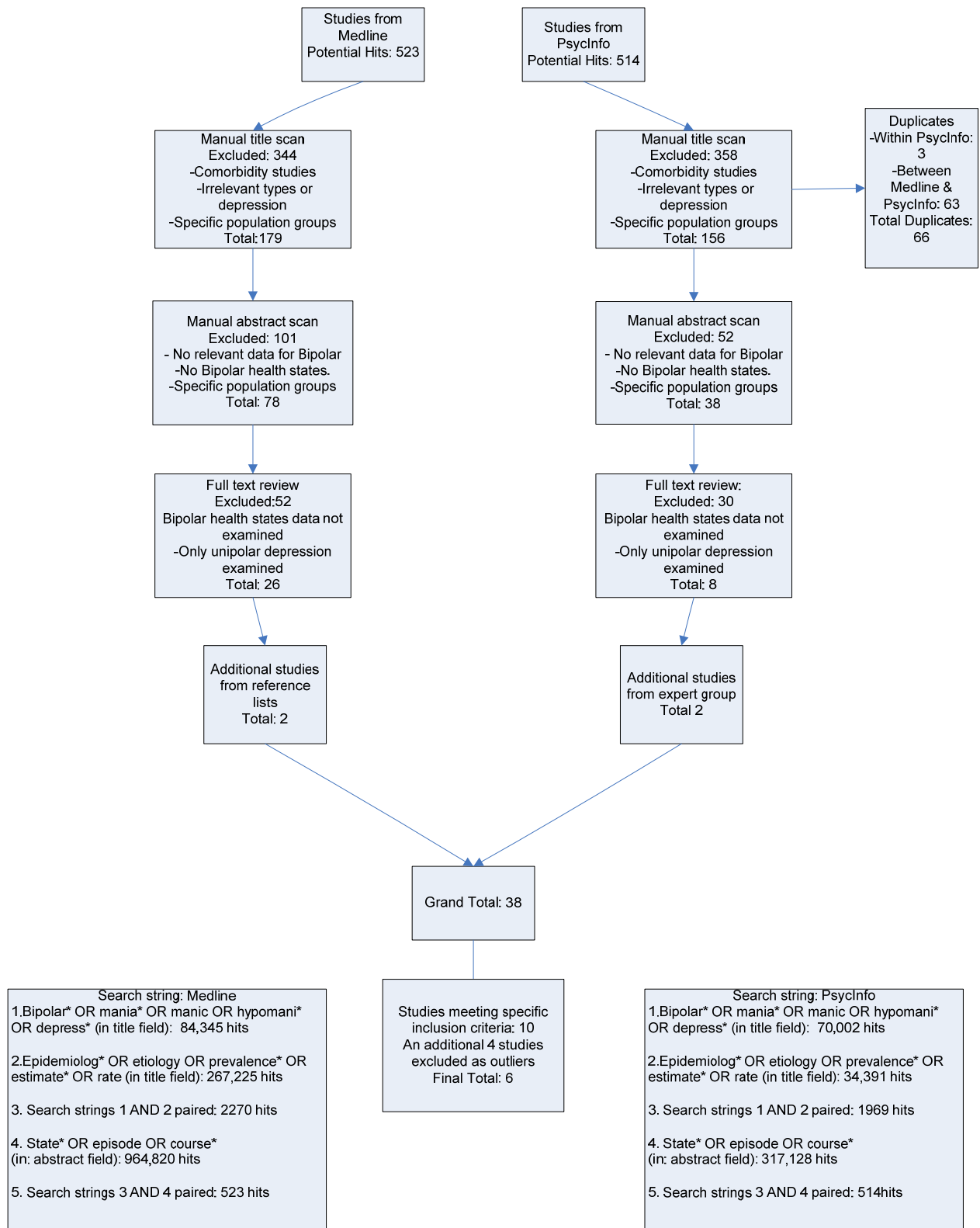

Supplement: Additional file 1 — Literature search strategy for bipolar disorder health states. [file 1478-7954-10-16-S1.pdf]
