# Supplementary material for: Health states for schizophrenia and bipolar disorder within the Global Burden of Disease 2010 Study
Source: Popul Health Metr. 2012 Aug 22;10:16. doi: 10.1186/1478-7954-10-16 (PMC3490927; doi:10.1186/1478-7954-10-16)
Supplement: Additional file 2 — Quality Index criteria. [file 1478-7954-10-16-S2.pdf]

Additional file 2.

Quality Index criteria

1. **Were the target population and the observation period well defined?**
   Yes = 1
   No = 0

2. **Diagnostic criteria**
   Use of diagnostic system reported (DSM, ICD, RDC) = 1
   Own system /symptoms described/no system/not specified = 0

3. **Method of case ascertainment**
   Community survey/multiple institutions = 2
   Inpatient/inpatients and outpatients/case registers = 1
   Not specified = 0

4. **Administration of measurement protocol**
   Administered interview = 3
   Systematic case note review = 2
   Chart diagnosis/case records = 1
   Not specified = 0

5. **Catchment Area**
   Broadly representative (national or multi-site survey) = 2
   Small area/not representative (single community, single university) = 1
   Convenience sampling/ other (primary care sample/treatment group) = 0

6. **Prevalence measure**
   Point prevalence (e.g. one month) = 2
   12-month prevalence = 1
   Lifetime prevalence = 0

Maximum Score = 11
